# Reading Notices

**Published:** 1910-11

**Authors:** 


### READING NOTICES

### DOCTOR REMEMBER.

DIOVIBURNIA is the most efficient Uterine Tonic, Alterative, Anti-Spasmodic and Anodyne, indicated in Anemia, Parturition, Leucorrhea, Menorrhagia, Subinvolution, Dysmenorrhea, Miscarriage, Vesical Tenesmus, Diarrhea, Dysentery, Chlorosis, Ovaritis, Cholera Morbus, Climacteric Diseases, Endometritis, Pterine Engorgement, Metrorrhagia, Prolapus Uteri, Threatened Abortion, Puerpal Convulsions, Vomiting of Pregnancy, Rela Condition of the Uterus.

NEUROSINE is the Standard Neurotic, Anodyne and Hypnotic. Contains no Opium, Morphine, Chloral or other deleterious drugs. Indicated in All Nuroses, Migrane, Hysteria, Spermatorrhea, Uterine Congestion, Shingles, Opium Habit, Chorea, Neuralgia, Asthma, Neurasthenia, Herpes, Delirium Tremens, Whooping Cough, Sea-Sickness and All Reflex and Convulsive Neuroses.

### THE TRUE NERVE CALMATIVE.

The Remedy PAR EXCELLENCE in Restlessness of Fevers, Producing Natural Sleep. NEUROSINE is Almost a Specific in Epilepsy.

---

### AN UNEXCELLED COMBINATION.

Two parts of Dioviburnia to One part of Neurosine is par excellent in Hysteria, Eclampsia, Melancholia, Female Neurosis,

Uterine Congestion, Ovarian Neuralgias, an Efficient Diuretic, Asthma Sexualis, Uterine Irritability, Lumbago, Migraine, Menopause, Menstrual Colic, Anemic Nervousness, Nervous Prostration, Reflex Cough, Delayed Catamenia, Non-Descriptive Cases, Subacute Rheumatism, Relieves all False Pains, Rheumatic, Sciatic Pains, Neurasthenia from Uterine Diseases.

---

### TO RELIEVE THE EFFECT OF SOLAR HEAT.

Direct exposure to the sun's rays; employment in or living in hot and poorly ventilated offices, workshops or rooms, are among prolific causes of headache in summer-time, as well as of heat exhaustion and sun-stroke. For these headaches and for the nausea which often accompanies them, antikamnia tablets will be found to afford prompt relief, and can be safely given. Insomnia from solar heat is readily overcome by one or two antikamnia tablets at supper time, and again before retiring. If these conditions are partly dependent upon a disordered stomach, two tablets with fifteen or twenty drops of aromatic spirits of ammonia, well diluted, are advisable. For the pain following sun or heat-stroke, antikamnia in doses of one or two tablets every two or three hours will produce the ease and rest necessary to complete recovery. As a preventive of and cure for nausea while traveling by railroad or steamboat, and for genuine *mal de mer,* or sea sickness, antikamnia tablets are unsurpassed.

---

### ONE COMMON LUNG BACILLUS.

It is quite generally accepted that pulmonary tuberculosis is caused by a bacillus. Coughs, colds, lagrippe and bronchitis come and go, even if we canot exhibit them as entities under the miscroscope. It would indeed be a fortunate thing if there were one common lung bacillus, the destruction of which would remove the cause of all respiratory affections. But under the present condition of things we can only meet indications, treat

